# Epithelial Thickness Changes After Descemet Membrane Endothelial Keratoplasty (DMEK): An Observational Study

**DOI:** 10.3390/jcm15051984

**Published:** 2026-03-05

**Authors:** Issac Levy, Lea Habib, Stephen Morgan, Ritika Mukhija, Mayank A. Nanavaty

**Affiliations:** 1 Sussex Eye Hospital, University Hospitals Sussex NHS Foundation Trust, Eastern Road, Brighton BN2 5BF, UK; 2 Brighton & Sussex Medical School, University of Sussex, Falmer, Brighton BN2 5BF, UK

**Keywords:** epithelial thickness, DMEK, corneal epithelium

## Abstract

**Aims**: The aim of this study is to characterise corneal epithelial thickness profiles after Descemet membrane endothelial keratoplasty (DMEK) and compare it with healthy controls, focusing on inferior–superior (I–S) epithelial thickness differences and their relationship with age. **Methods**: This single-centre observational study included 36 post-DMEK eyes with at least 6 months’ follow-up and 36 healthy control eyes. High-resolution spectral-domain anterior segment OCT maps were analysed for central epithelial thickness (CET, defined as the mean epithelial thickness within the central 2 mm zone [E2.0]) and peripheral sectors to derive inferior (E–I) and superior (E–S) values (between 2 and 7 mm), with the I–S difference computed at a 3 mm radius; group comparisons used t-tests and correlations used Pearson’s r (α = 0.05). Central corneal thickness (CCT) was also compared between groups. Results: Post-DMEK eyes had significantly lower mean CCT than controls (525.7 ± 98.4 μm vs. 544.71 ± 27.8 μm, *p* = 0.04). Central epithelial thickness did not differ between groups (post-DMEK 53.7 ± 5.5 μm vs. controls 52.7 ± 3.3 μm, *p* = 0.62), but the I–S epithelial difference was greater after DMEK (5.9 ± 4.3 μm) than controls (3.0 ± 2.2 μm, *p* < 0.01), indicating a more pronounced inferior thickening pattern. Age showed no significant relationship with epithelial thickness in controls, and only very weak or non-significant correlations with central thickness and I–S difference in post-DMEK eyes, indicating no clinically meaningful age effect postoperatively. **Conclusions**: DMEK restores central epithelial thickness to values comparable to normal eyes, while accentuating the physiologic inferior–superior epithelial gradient, consistent with localised postoperative epithelial remodelling rather than global epithelial thickening or thinning. Corneal stromal remodelling may result in lower CCT post-DMEK versus controls, and age does not meaningfully influence epithelial distribution after surgery.

## 1. Introduction

The corneal epithelium, a non-keratinised stratified squamous epithelium, forms the cornea’s outermost layer and serves as the primary interface between the external environment and the optical apparatus of the eye. This highly specialised tissue consists of 5–7 cell layers organised in a tightly regulated architecture: a single layer of columnar basal cells attached to the basement membrane, 2–3 layers of polygonal wing cells, and 2–3 layers of flattened superficial cells [1]. The epithelium measures approximately 54 ± 5 μm centrally, being thickest at the centre and gradually becoming thinner towards the periphery [1]. The basement membrane, ranging from 0.4 to 0.55 μm in thickness, comprises the lamina lucida and lamina densa and contains type IV collagen and laminin as its primary structural components [2]. Basal epithelial cells maintain attachment to this basement membrane through specialised structures called hemidesmosomes, which extend into the corneal stroma via anchoring plaques composed of type I collagen, ensuring mechanical stability of the epithelial layer [2].

The corneal epithelium exhibits remarkable functional complexity beyond its structural role. As the outermost layer, in conjunction with the tear film, it serves as a highly important optical interface, and its regularity is essential for maintaining a smooth refractive surface with minimum aberrations [3]. The epithelium also provides a crucial barrier function, preventing pathogen entry and maintaining corneal homeostasis. Notably, the corneal epithelium possesses the highest density of sensory nerve innervation of any tissue in the human body‚ approximately 400 times greater than the epidermis‚ which plays essential roles in supporting epithelial metabolism, regulating cell proliferation and differentiation, and coordinating wound healing responses. This dense neural network contains neuropeptides such as substance P, calcitonin gene-related peptide, and vasoactive intestinal peptide, which are critical for epithelial maintenance and renewal [2]. Furthermore, the corneal epithelium exhibits remarkable regenerative capacity, with complete epithelial turnover occurring approximately every 7–10 days in humans, allowing brisk restoration of this outer layer following injury and surgery. Epithelial remodelling following laser refractive surgery, for example, is a well-documented phenomenon that can significantly influence postoperative outcomes [3].

Corneal homeostasis is maintained through intricate intercellular signalling networks, particularly the bidirectional crosstalk between limbal epithelial and stromal cells. Limbal stem cells residing in the palisades of Vogt are regulated by their niche microenvironment, which includes mesenchymal stromal cells that secrete growth factors, cytokines, and extracellular vesicles influencing stem cell quiescence, activation, and differentiation. This epithelial–stromal interaction modulates wound healing responses and regulates stem cell phenotypes that are directly associated with epithelial thickness homeostasis. Disruption of these signalling pathways, as may occur in endothelial dystrophies and following surgical intervention, can alter epithelial regeneration patterns and thickness distribution [4].

In normal corneas, epithelial thickness exhibits a characteristic topographic distribution pattern. Previous studies using high-frequency ultrasound biomicroscopy and spectral-domain optical coherence tomography (SD-OCT) have consistently demonstrated that the inferior and nasal epithelium is slightly thicker than the superior and temporal regions [5,6]. This asymmetry, typically quantified as an inferior–superior (I–S) difference of approximately 2–3 μm in healthy eyes, has been attributed to mechanical factors, particularly the more frequent friction of the upper eyelid and the temporal area during blinking [5,6]. This physiologic thickness gradient appears to represent an adaptive response to external mechanical forces and may serve to maintain optimal optical quality across the corneal surface [7].

Fuchs’s Endothelial Dystrophy (FED) represents a progressive bilateral corneal disorder characterised by accelerated endothelial cell loss, leading to profound alterations in corneal architecture and function. The pathophysiology of FED is multifactorial, involving genetic predisposition‚ particularly trinucleotide repeat expansions in the TCF4 gene identified in nearly 70% of late-onset cases‚ oxidative stress, mitochondrial dysfunction, and abnormal extracellular matrix deposition [8]. Early-onset FED has been associated with mutations in ZEB1 and COL8A2 genes, which affect endothelial cell phenotype and Descemet membrane composition [8]. The hallmark of FED is the formation of guttae‚ excrescences of abnormal collagen and extracellular matrix components deposited by dysfunctional endothelial cells onto the posterior Descemet membrane [9]. These guttae, which typically originate centrally and radiate peripherally as the disease progresses, are accompanied by progressive reduction in endothelial cell density (ECD), with normal values of 2500–3000 cells/mm, declining to below 1500 cells/mm in early disease and below 500 cells/mm in advanced stages [8].

Progressive endothelial cell loss in FED fundamentally disrupts the corneal “pump-leak” mechanism, which is essential for maintaining corneal transparency. The endothelial monolayer functions as an active pump through Na^+^/K^+^-ATPase, bicarbonate–chloride transporters, and carbonic anhydrase, which collectively maintain the corneal stroma at approximately 78% hydration by counteracting the inherent swelling pressure generated by stromal glycosaminoglycans (the “leak”) [10]. When endothelial pump function becomes inadequate, the resulting fluid accumulation leads initially to stromal oedema, manifested as increased corneal thickness and decreased transparency, followed by epithelial oedema in more advanced stages. The compromised endothelial barrier allows excessive fluid entry into the stroma, overwhelming the epithelial layer’s compensatory mechanisms.

Four stages of FED have been described in the literature, with each stage exhibiting progressively severe abnormalities in corneal architecture [11,12]. In early stages (Stages 1–2), guttae are present but corneal oedema remains minimal or absent. Stage 3 disease is characterised by stromal oedema with increased central corneal thickness, while Stage 4 demonstrates both stromal and epithelial oedema. The epithelium in FED shows histological features of chronic oedema, with basal epithelial cells appearing pale and swollen [8,9]. In advanced cases, superficial epithelial cells may lift off, forming bullae or blebs that cause significant pain and visual impairment [8,9]. Microcystic changes and irregular, oedematous superficial epithelial cells have been observed, along with thickening of the epithelial basement membrane and abnormal basement membrane deposition within the epithelium [13]. In end-stage FED, subepithelial fibrosis and scarring occur due to long-standing oedema and cellular damage [8].

Descemet membrane endothelial keratoplasty (DMEK) has emerged as the gold standard treatment for endothelial decompensation associated with FED and bullous keratopathy, offering superior visual outcomes compared to earlier techniques such as penetrating keratoplasty (PK) and Descemet stripping automated endothelial keratoplasty (DSAEK) [14]. The procedure involves selective replacement of the diseased Descemet membrane and endothelium with a thin donor graft containing only these two layers, without the stromal component present in DSAEK [14]. Postoperative effects of DMEK include a marked reduction in central corneal thickness resulting from newly functioning transplanted endothelial cells that dehydrate the oedematous cornea, faster visual rehabilitation, and lower rates of immunologic rejection [14,15]. The changes in overall corneal and stromal thickness following DMEK have been extensively documented, with most studies reporting stabilisation of corneal thickness within 1–6 months postoperatively [16,17].

However, our understanding of changes to the epithelial layer specifically after DMEK remains remarkably limited. To our knowledge, only one published study has systematically investigated the effects of DMEK on epithelial thickness in patients with FED. Storp et al. [18] reported that post-DMEK corneal epithelial thickness decreased to a thickness comparable to that of the control group, with significant reduction observed in central, paracentral, and mid-peripheral zones. Their study emphasised that structural alterations in FED extend beyond the corneal stroma to involve the epithelial layer, and that DMEK induces normalisation of epithelial thickness. However, this study did not specifically examine epithelial thickness distribution patterns or the inferior–superior gradient in post-DMEK eyes. Furthermore, there are no studies examining the corneal epithelial changes specifically in Stage 2 and 3 FED patients undergoing DMEK [11,12].

Understanding epithelial thickness changes after DMEK has important clinical implications. Epithelial remodelling is recognised as a compensatory mechanism that can mask underlying stromal irregularities and influence optical outcomes [19]. The magnitude and pattern of epithelial changes may reflect the degree of corneal healing, biomechanical adaptation, and restoration of normal physiologic gradients [20]. Additionally, epithelial thickness mapping has emerged as a valuable diagnostic tool for detecting various corneal pathologies, including keratoconus, ectasia, and epithelial basement membrane dystrophy [21]. Whether the epithelial changes induced by chronic endothelial disease and subsequent DMEK surgery differ from normal physiologic patterns remains an open question with potential implications for predicting visual outcomes and understanding corneal wound healing.

The primary objective of this study is to investigate the epithelial thickness profiles in post-DMEK eyes with Stage 2 and Stage 3 FED [12] and compare them with healthy control eyes, with a specific focus on the inferior–superior epithelial thickness gradient. Secondary objectives include assessing the correlation between age and epithelial thickness distribution patterns in both groups and evaluating central corneal thickness differences between post-DMEK and control eyes to understand the contribution of stromal versus epithelial changes to overall corneal thickness.

## 2. Methods

### Study Design

This retrospective observational study took place at a single tertiary care centre (Sussex Eye Hospital, University Hospitals Sussex NHS Foundation Trust, Brighton, UK). The hospital audit committee approved the study (audit number 2558), which was conducted in accordance with the principles outlined in the Declaration of Helsinki. The fully anonymised data was collected from our electronic system between October 2024 and September 2025 including patients who underwent DMEK surgery in the Sussex Eye Hospital between February 2021 and December 2024, using their most recent follow-up corneal epithelial map scan. Inclusion criteria for the post-DMEK group were patients with Stages 2 and 3 of FED [22], who underwent successful surgery without complications, and had a minimum follow-up period of six months to allow for epithelial remodelling, with availability of anterior segment OCT data, and the absence of other ocular comorbidities such as glaucoma, lid abnormalities, previous ocular surgeries, etc. Exclusion criteria were patients who underwent DMEK for corneal decompensation from causes other than FED and eyes with any intraoperative or postoperative complications such as hyphaema, secondary procedures, graft detachment, rejection, or failure. The control patients were selected from patients who were referred to our clinic for various ocular complaints or pathologies unrelated to the cornea, or fellow eyes of patients with a unilateral corneal scar, who underwent a corneal map scan and were found to have healthy corneas upon examination. Individuals with a normal corneal epithelial map were included in the study, and both eyes were eligible for inclusion; however, if one eye exhibited a pathological map, it was excluded.

All individuals in this study underwent DMEK using a standardised protocol consistently applied in our department, as described in an earlier publication [23]. On the day of surgery, the surgeon prepared each corneal graft in the operating theatre using a modified manual dissection technique based on the Melles approach. Only donor grafts with an endothelial cell density exceeding 2200 cells/mm^2^ were selected for DMEK preparation within our unit. After trephining the graft to 7.75 mm and placing a triangular orientation marker, the tissue was loaded into a sterile, single-use Geuder injector cartridge (Geuder AG, Heidelberg, Germany) just before transplantation. Once introduced and correctly oriented in the recipient’s anterior chamber, the graft was unfolded and secured with a complete chamber fill of either air or 50% sulphur hexafluoride (SF6) gas. The primary surgical incision and two paracenteses were closed with 10-0 nylon sutures. In addition, a vent paracentesis was created at the 5 o’clock position to allow controlled release of aqueous and air/gas postoperatively at the slit lamp, as previously reported [23,24].

Standardised postoperative care included review in the ward 1–2 h post-surgery (POD-0), during which a controlled release of air or gas was performed through the vent paracentesis to restore anterior chamber depth while maintaining a large tamponade bubble (around 80% chamber fill). On postoperative day 1 (POD-1), further aqueous was released via the same site to ensure the tamponade bubble now occupied approximately 70% of the chamber. This adjustment is especially important with SF6 owing to expected early expansion. This approach provides sufficient space for aqueous production as the air bubble gradually diminishes. Both interventions were carried out at the slit lamp under topical anaesthesia (proxymetacaine and povidone–iodine minims), with full aseptic precautions. Intraocular pressure was not routinely measured at the end of surgery or during gas release, as readings can be unreliable; instead, resolution of epithelial oedema and normalisation of anterior chamber depth were used as clinical proxies. Patients were seen again one week after surgery. They were encouraged to lie supine for the first three days, though strict posturing was not enforced [23].

**Figure 1:**
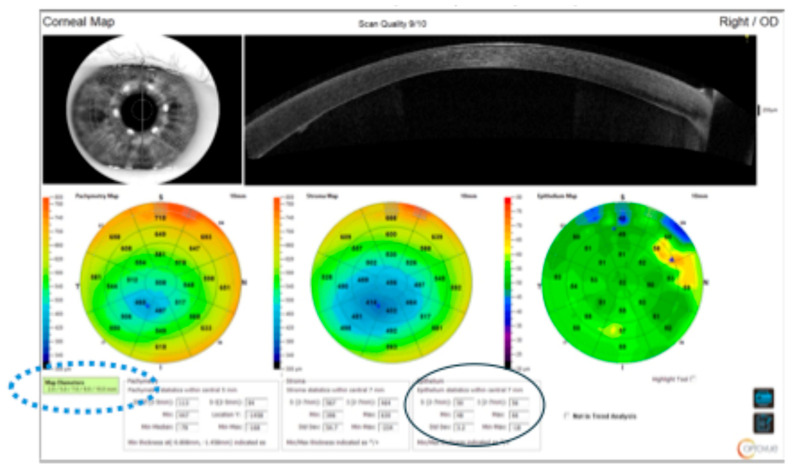
Epithelial thickness map in post-DMEK eye. Circle highlights the superior (E–S) and inferior (E–I) epithelial thickness in 2–7 mm zone. The dotted circle shows the zone of central 2 mm, 5 mm, 7 mm, 9 mm, 10 mm.

Initial postoperative therapy comprised a combination antibiotic–steroid eye drop (topical tobramycin and dexamethasone) administered four times daily for four weeks, along with cycloplegics (cyclopentolate 1% three times daily) and an oral IOP-lowering agent (acetazolamide SR 250 mg twice daily) for one week. Sutures were typically removed within four weeks, at which point patients switched to a steroid-only regimen (Loteprednol etabonate, reducing from four times daily over several months to once daily by two years) [23]. Corneal epithelial thickness was subsequently assessed using high-resolution spectral-domain anterior segment OCT (Solix, Visionix, France), and parameters were analysed as part of this study as follows (Figure 1):

- E2.0: Epithelial thickness in the central 2 mm or central epithelial thickness (CET);
- E–S (2–7 mm): Epithelial thickness in the superior cornea (between 2 mm and 7 mm zone);
- E–I (2–7 mm): Epithelial thickness in the inferior cornea (between 2 mm and 7 mm zone).

Epithelial thickness measurements were taken at a 10 mm radius from the corneal centre. The I–S epithelial thickness difference was calculated for each eye.

We planned a two-sample comparison of mean corneal epithelial thickness (OCT-derived) between healthy control eyes and eyes after DMEK surgery using a two-sided t-test with α = 0.05 and 90% power. A priori sample size was calculated for a two-sample comparison of mean corneal epithelial thickness between groups using a two-sided independent t-test. The following parameters were assumed: within-group standard deviation (σ) = 5 µm (based on published OCT-based epithelial thickness data [18,25]; minimal clinically important difference (δ) = 3.2 µm; significance level (α) = 0.05; statistical power (1 − β) = 90%. Using the standard formula for two independent means with equal allocation: n per group = 2 × (Z_1__−_α/_2_ + Z_1__−_β)^2^ × σ^2^/δ^2^, the required sample size was calculated as 36 eyes per group. The final enrolled sample of 36 eyes per group meets this requirement. Thus, enrolling 36 eyes in each group provides at least 90% power to detect clinically meaningful differences of approximately 3.2 μm (or larger) under realistic variability, while balancing feasibility and precision.

Data normality was assessed using the Shapiro–Wilk test. Between-group comparisons of continuous variables (CET, I–S difference, CCT) were performed using independent two-sample t-tests (two-tailed). Welch’s correction was applied when Levene’s test indicated unequal variances. To account for the age difference between groups, analysis of covariance (ANCOVA) was performed with age as a covariate for all primary outcomes. Pearson’s correlation coefficient (r) was used to assess relationships between age and epithelial parameters. Effect sizes were calculated as Cohen’s d with 95% confidence intervals for all primary comparisons. Exact *p*-values are reported throughout; significance was set at α = 0.05. No formal correction for multiple comparisons was applied, as the primary outcomes were pre-specified; this is acknowledged as a limitation. A sensitivity analysis including time since surgery as a covariate was also performed.

## 3. Results

This study included 36 eyes (36 patients) with Stage 2 and 3 FED patients who underwent DMEK surgery between February 2021 and December 2024 and a control group of 36 healthy eyes from 19 healthy individuals. The mean age (years) in the post-DMEK group was 71.4 ± 12.1, and in the control group, it was 45.5 ± 14.6 (*p* < 0.01). All post-DMEK patients were pseudophakic and had received 50% SF_6_ gas tamponade. None had concurrent glaucoma, ocular surface disease, or prior ocular surgery other than cataract extraction. There were no intraoperative or postoperative complications in any eyes in the post-DMEK group, with a median follow-up period of 24 months (IQR: 12–54 months; mean 35.5 ± 36.6 months) (Table 1) (Supplementary Table S1).

Mean CCT was significantly lower after DMEK compared to controls (525.7 ± 98.4 µm vs. 544.7 ± 27.8 µm, *p* = 0.04, Cohen’s d = 0.26, 95% CI of difference: −37.1 to −0.9 µm). After age adjustment (ANCOVA), the difference remained significant (adjusted *p* = 0.04).

### 3.1. Epithelial Thickness

**Figure 2:**
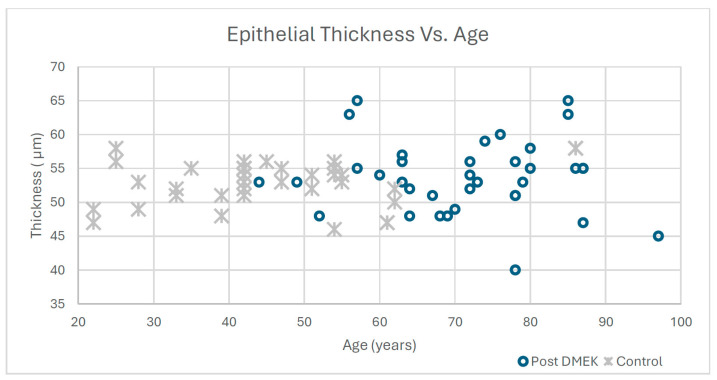
Correlation between Central Epithelial Thickness vs. Age in Post-DMEK Patients and Controls.

Table 1 presents the group demographics and summary epithelial thickness data (individual patient data is provided in Supplementary Table S1). No significant difference was found between the groups in mean CET (*p* = 0.62, Cohen’s d = 0.22), with values of 53.7 ± 5.5 µm in the post-DMEK group and 52.7 ± 3.3 µm in the control group. The I–S epithelial difference was significantly greater after DMEK (5.9 ± 4.3 µm) compared to 3.0 ± 2.2 µm in controls (*p* = 0.001, Cohen’s d = 0.85, 95% CI of difference: 1.3 to 4.5 µm). After age adjustment, the I–S difference remained significantly greater in the post-DMEK group (ANCOVA, adjusted *p* < 0.01). Supplementary Figure S1A,B shows the scatter plot between 1–S difference versus age and versus CCT. Figure 2 shows the correlation between the age of the patients and central epithelial thickness.

### 3.2. Correlation Between Age and I–S Thickness Difference

In the control group, Pearson’s correlation coefficient between age and CET was r = 0.12 (*p* = 0.62) and between age and I–S difference it was r = 0.32 (*p* = 0.57), indicating no significant relationship. In the post-DMEK group, the correlation between age and CET was r = −0.05 (*p* < 0.01) and between age and I–S difference it was r = 0.21 (*p* = 0.22). Although the age–CET correlation reached statistical significance, the coefficient of determination (R^2^ = 0.0025) indicates that age explains less than 1% of the variance in CET, rendering this association clinically negligible. These results indicate that age does not meaningfully affect the distribution of epithelial thickness. A sensitivity analysis including time since surgery as a covariate did not materially alter the above findings. Supplementary Figure S1A shows the scatter plot.

Supplementary Table S2 shows the epithelial parameters stratified by follow-up duration in post-DMEK eyes. A sensitivity analysis was performed in which time since surgery (months) was entered as a continuous covariate, along with age, in linear models for CET, I–S difference, and CCT in post-DMEK eyes. Time since surgery was not a significant predictor of CET (β close to 0, *p* > 0.2), I–S difference (*p* > 0.2), or CCT (*p* > 0.2), and inclusion of this covariate did not materially change the estimated between-group differences or their statistical significance. These results indicate that, within the observed range of follow-up (6–106 months), variation in time since surgery does not meaningfully influence epithelial thickness distribution or CCT after DMEK.

## 4. Discussion

This study demonstrates that DMEK surgery in patients with Stage 2 and 3 FED effectively restores CET to values comparable to healthy controls while significantly accentuating the physiologic inferior–superior epithelial thickness gradient.

Postoperative stromal and epithelial remodelling may also be influenced by altered intercellular signalling mediated by extracellular vesicles. Emerging evidence suggests that exosomes carrying miRNA and protein cargos play a pivotal role in epithelial–stromal communication, modulating wound healing, cell proliferation, and tissue regeneration in the cornea. These molecular signalling pathways may establish a mechanistic link between the imaging phenotypes observed in our study (i.e., the augmented I–S gradient) and underlying cellular processes driving post-DMEK remodelling [26].

Our central finding‚ that mean epithelial thickness after DMEK (53.7 ± 5.5 μm) does not differ significantly from controls (52.7 ± 3.3 μm)‚ confirms and extends the observations of Storp et al. [18], who reported that post-DMEK corneal epithelial thickness decreased to levels comparable to those of healthy individuals. This normalisation of central epithelial thickness represents a reversal of the epithelial thickening that occurs in FED due to chronic stromal and epithelial oedema. In untreated FED, particularly in Stages 3 and 4, the epithelium becomes thickened centrally and inferiorly, with histological features including pale, swollen basal cells, microcystic changes, and in advanced cases, bullous formation [8,9,25]. The restoration of endothelial pump function following DMEK resolves this pathologic epithelial thickening by eliminating the underlying stromal oedema that drives fluid accumulation in the epithelial layer.

The magnitude and time course of epithelial normalisation following DMEK appear to parallel the well-documented reduction in stromal thickness. Multiple studies have demonstrated that central corneal thickness decreases significantly within the first month after DMEK, reaching near-normal values by 3–6 months postoperatively [16,17,27]. Machalinska et al. [17] reported that CCT reduction after DMEK was noticeable from the first month and continued for up to 12 months, with the data suggesting that this reduction should be attributed, in part, to de-swelling of the corneal epithelium. Our study, with a mean follow-up of 35.5 months, captures the stable, long-term epithelial thickness achieved after this initial remodelling period. This temporal pattern suggests that epithelial remodelling after DMEK is an active process coordinated with stromal dehydration, rather than a passive response to thickness changes alone.

Reinstein et al. [5] conducted a seminal study characterising epithelial thickness in the normal cornea using very high-frequency digital ultrasound, finding a mean epithelial thickness of 53.4 ± 4.6 μm, with no statistically significant difference between right and left eyes. This value is remarkably consistent with both our control group (52.7 ± 3.3 μm) and our post-DMEK group (53.7 ± 5.5 μm), supporting the conclusion that DMEK achieves true normalisation of central epithelial thickness rather than producing an abnormal thinning or thickening pattern. The consistency of central epithelial thickness values across multiple studies using different measurement technologies (high-frequency ultrasound, spectral-domain OCT, swept-source OCT) also validates the reliability of modern epithelial thickness mapping techniques for clinical and research applications [18,28,29].

The most striking finding of our study is the significantly increased inferior–superior epithelial thickness difference in post-DMEK eyes (5.9 ± 4.3 μm) compared to controls (3.0± 2.2 μm, *p* < 0.01). This accentuation of the physiologic I–S gradient by approximately 100% represents a distinctive signature of post-DMEK corneal architecture. While the inferior epithelium remains thicker than the superior epithelium‚ consistent with the normal pattern observed in healthy eyes‚ the magnitude of this difference is nearly doubled after DMEK surgery. This finding suggests that DMEK does not simply restore pre-disease epithelial architecture but rather produces a modified epithelial topography that reflects both the resolution of pathologic changes and the emergence of new adaptive patterns.

Several mechanisms may contribute to this augmented I–S gradient. First, localised epithelial remodelling in response to residual topographic irregularities in the anterior stromal surface may drive differential epithelial thickening. The epithelium has a well-established capacity to compensate for underlying stromal irregularities by modulating thickness, effectively “smoothing out” variations in stromal topography to maintain optical quality [20,28,30]. Studies of epithelial remodelling after refractive surgery have demonstrated that the magnitude of epithelial thickness changes correlates with the rate of change in corneal curvature, with steeper curvature transitions eliciting greater epithelial remodelling [19]. Following DMEK, subtle biomechanical changes in the cornea‚ including the interface between the recipient peripheral cornea and the central donor graft‚ may create localised zones of altered curvature that drive differential epithelial thickening, particularly in the inferior cornea, where gravitational and mechanical forces may be most pronounced.

Second, biomechanical factors related to the surgical alteration of corneal structure may influence epithelial distribution. DMEK fundamentally changes the posterior corneal architecture by replacing the diseased Descemet membrane and endothelium with a thinner donor graft, typically 8.0 mm in diameter, that must integrate with the peripheral recipient cornea. Hayashi et al. [27] demonstrated that eyes after DMEK exhibit altered posterior corneal curvature and higher-order aberrations compared with normal eyes, even 12 months postoperatively, despite similar central corneal thickness. These persistent structural differences may translate into differential mechanical stress patterns across the epithelial surface, with the inferior cornea potentially experiencing greater stress due to gravitational effects, eyelid pressure during blinking, and the natural inferior tear meniscus. The epithelium may respond to these altered biomechanical conditions by increasing thickness in the inferior region to maintain mechanical stability and optimal optical performance.

Third, wound healing processes and nerve regeneration patterns after DMEK may contribute to the augmented I–S gradient. Corneal epithelial wound healing is orchestrated by multiple growth factors and signalling molecules, including epidermal growth factor (EGF), nerve growth factor (NGF), hepatocyte growth factor (HGF), and substance P [31,32]. The expression and distribution of these factors may vary topographically across the corneal surface during the healing period after DMEK. Moreover, corneal nerve regeneration following corneal surgery exhibits specific temporal and spatial patterns. Studies of nerve regeneration after various keratoplasty techniques have demonstrated that reinnervation progresses centripetally from the peripheral margins, with sub-basal nerve density gradually recovering over 6–24 months postoperatively [33,34]. Since nerve density influences epithelial cell proliferation, migration, and metabolism [2], regional variations in nerve regeneration patterns could contribute to differential epithelial thickness across the corneal surface. The inferior cornea, which maintains greater limbal proximity and potentially earlier reinnervation, may exhibit enhanced epithelial proliferative capacity, leading to greater thickness compared to the superior region.

Fourth, the surgical technique itself may contribute to regional epithelial variations. During DMEK surgery, the descemetorhexis (removal of the recipient’s diseased Descemet membrane and endothelium) creates a zone of endothelial denudation that must be covered by the donor graft [35]. If the descemetorhexis is larger than the graft, peripheral areas remain denuded, requiring endothelial cell migration from the graft edge to repopulate these regions [27]. This migration pattern may create subtle regional variations in endothelial cell density and pump function, which, in turn, could influence overlying epithelial thickness by altering local hydration control. The inferior cornea, potentially receiving endothelial cell coverage later or at lower density compared to the central graft area, might develop compensatory epithelial thickening to maintain optimal corneal function.

The absence of a significant correlation between age and epithelial thickness difference in both control and post-DMEK groups (control: r = 0.32, *p* = 0.57; post-DMEK: r = 0.21, *p* = 0.22) aligns with findings from several previous studies and has important clinical implications. Reinstein et al. [5] and Colakoglu and Cosar [36] reported no significant age-related changes in central epithelial thickness in healthy eyes, although some studies have found mild thinning of central epithelial thickness with advancing age. The lack of age correlation in our post-DMEK cohort suggests that epithelial remodelling after surgery is primarily driven by local biomechanical and healing factors rather than by age-dependent cellular changes. This finding is clinically reassuring, as it indicates that older patients‚ who comprise the majority of FED cases requiring DMEK‚ can be expected to achieve epithelial normalisation comparable to younger patients. The independence of epithelial remodelling from age also suggests that cellular mechanisms governing epithelial thickness regulation remain intact and responsive even in older corneas that have undergone significant pathologic changes and surgical intervention.

Our finding of significantly lower mean central corneal thickness in post-DMEK eyes (525.7 ± 98.4 μm) compared to controls (544.7 ± 27.8 μm, *p* = 0.04) warrants cautious interpretation given the significant age difference between groups (mean 71.4 vs. 45.5 years, *p* < 0.01). Although ANCOVA with age as a covariate confirmed that the CCT difference remained significant (adjusted *p* = 0.04), several alternative explanations should be considered. First, although most studies report no significant age-related change in CCT in healthy adults [36], subtle age-related stromal thinning cannot be entirely excluded. Second, the large standard deviation in post-DMEK CCT (98.4 µm) indicates considerable inter-individual variability that may reflect differences in disease duration, severity, and healing responses. Third, measurement variability and selection bias in the control group may contribute to observed differences. Nevertheless, our findings are consistent with previous reports by Storp et al. [18] and Hayashi et al. [27], who documented reduced stromal thickness after DMEK using age-matched or similar control populations, supporting the interpretation that the observed CCT difference at least partly reflects chronic stromal remodelling from long-standing endothelial disease.

These persistent thickness alterations may result from irreversible stromal remodelling associated with chronic endothelial dysfunction. Long-standing FED involves not only fluid accumulation but also alterations in stromal collagen architecture, keratocyte density and function, and extracellular matrix composition [8,37]. Studies using confocal microscopy have documented reduced anterior keratocyte density and fibroblastic transformation of stressed keratocytes in the stroma of FED patients [25]. Even after successful DMEK restores endothelial pump function and resolves oedema, these structural changes may persist, resulting in a slightly thinner cornea compared to eyes that never experienced endothelial disease. The relatively large standard deviation in post-DMEK CCT (98.4 μm) compared to controls (27.8 μm) also suggests considerable variability in stromal remodelling patterns among individual patients, possibly reflecting differences in disease duration, severity, and individual healing responses.

An important consideration in interpreting our CCT findings is the significant age difference between the post-DMEK group (mean 71.4 ± 12.1 years) and the control group (mean 45.5 ± 14.6 years). While studies have shown that central corneal thickness does not significantly change with age in healthy adults [36], this age disparity remains a potential confounding factor. Future studies with age-matched control groups would strengthen conclusions regarding stromal remodelling after DMEK. Nevertheless, the consistency of our findings with other reports using various control populations suggests that the observed CCT difference reflects true post-DMEK structural changes rather than age-related effects.

The clinical implications of our findings extend to several areas of ophthalmic practice. First, understanding that DMEK normalises central epithelial thickness while accentuating the I–S gradient may help clinicians interpret postoperative anterior segment OCT scans and distinguish normal postoperative patterns from pathologic changes. Epithelial thickness mapping is increasingly used to detect early corneal ectasia, monitor disease progression in keratoconus, and identify subclinical corneal abnormalities before refractive surgery [21,30]. Establishing normative post-DMEK epithelial patterns will enable more accurate interpretation of these diagnostic scans in patients who have undergone corneal transplantation. Second, the augmented I–S gradient after DMEK may have optical consequences that influence visual quality outcomes. While our study did not measure visual acuity or higher-order aberrations, previous research has demonstrated correlations between epithelial thickness variations and corneal aberrations [20,27]. Future studies correlating post-DMEK epithelial topography with patient-reported visual quality metrics and objective aberrometry measurements would provide valuable insights into the functional significance of these epithelial changes. Third, our findings have implications for surgical planning and postoperative management. The observation that epithelial thickness normalises after DMEK but exhibits an altered distribution pattern suggests that adequate time must be allowed for epithelial remodelling to stabilise before performing additional procedures such as cataract surgery or refractive interventions in post-DMEK eyes. Studies of epithelial remodelling after refractive surgery have shown that stabilisation typically occurs between 3 and 6 months postoperatively, with some procedures requiring up to 6–12 months for complete epithelial adaptation [38,39]. Our study, with an average follow-up exceeding 35 months, captures the long-term stable epithelial pattern after DMEK. Clinicians should be aware that epithelial remodelling may continue for several months postoperatively and that measurements taken in the early postoperative period may not reflect the final epithelial architecture. Fourth, epithelial thickness monitoring may serve as a biomarker for graft function and corneal health after DMEK. Long-term studies have shown that DMEK provides excellent graft survival rates, with 5-year survival ranging from 83 to 96% and a 10-year survival of approximately 75–92% depending on study populations and definitions of graft failure [14,40,41]. However, gradual endothelial cell loss continues after DMEK, with 10-year probability of maintaining endothelial cell density above 1000 cells/mm ≤ reported as only 3–8% [14]. As endothelial function gradually declines, subtle changes in epithelial thickness might serve as an early indicator of impending decompensation. Serial epithelial thickness mapping in post-DMEK patients could potentially identify early graft dysfunction before clinical oedema becomes apparent, enabling timely intervention. Further research is needed to determine whether progressive epithelial thickening or altered thickness distribution patterns can predict graft failure or reduced endothelial reserve.

The use of spectral-domain optical coherence tomography (SD-OCT) for epithelial thickness measurements in our study warrants discussion. SD-OCT utilises light with a wavelength of approximately 840 nm and achieves axial resolution of approximately 5 μm in tissue, making it well-suited for imaging superficial structures like the corneal epithelium [42,43]. Compared to swept-source OCT (SS-OCT), which uses longer wavelengths (~1050 nm) and provides deeper tissue penetration, SD-OCT offers superior resolution and contrast for anterior segment structures, including the epithelium [42,43]. Multiple studies have validated the repeatability and reproducibility of SD-OCT for epithelial thickness mapping, with coefficients of variation typically less than 6% for epithelial measurements [29,43]. The Solix SD-OCT system used in our study provides automated segmentation algorithms and generates comprehensive epithelial thickness maps across a wide field of view, enabling detailed analysis of regional thickness variations.

Our study has several strengths that enhance confidence in the findings. First, the relatively large sample size (36 eyes per group) and long mean follow-up period (35.5 months) provide robust data on stable, long-term epithelial patterns after DMEK. Second, the use of standardised surgical technique performed at a single centre by experienced surgeons minimises technical variability that could confound results. All patients received identical postoperative care protocols, donor tissue preparation, and graft dimensions (7.75 mm), ensuring consistency. Third, excluding eyes with intra- or postoperative complications eliminates the confounding effects of rebubbling, graft detachment, rejection, or failure on epithelial thickness measurements. Fourth, the use of high-resolution SD-OCT with automated analysis provides objective, reproducible measurements free from observer bias.

Several limitations must be acknowledged. First, the significant age difference between the post-DMEK group (mean 71.4 years) and controls (mean 45.5 years) represents a major potential confounding factor. Although age-adjusted analyses (ANCOVA) confirmed the robustness of our primary findings, future studies with age-matched control groups are essential to definitively exclude age-related confounding. Second, the absence of preoperative epithelial thickness measurements is a significant limitation, as it precludes direct quantification of DMEK-induced epithelial remodelling and limits attribution of observed patterns solely to the surgical intervention. Future prospective studies with serial preoperative and postoperative measurements are needed to address this gap. Third, the retrospective design and non-randomised patient selection introduce potential selection biases. Fourth, the wide follow-up distribution (median 24 months, IQR 12–54 months) may introduce variability, although sensitivity analyses with time since surgery as a covariate showed no significant effect on outcomes. Fifth, our study included only patients with Stage 2 and 3 FED who underwent successful DMEK without complications. Sixth, we did not apply a formal correction for multiple comparisons; although the primary outcomes were pre-specified, the possibility of type I error from multiple testing cannot be excluded. Seventh, we did not correlate epithelial thickness patterns with visual outcomes, higher-order aberrations, or patient-reported visual quality measures. Eighth, our study did not examine potential factors that might influence epithelial remodelling after DMEK, such as donor tissue characteristics (endothelial cell density, donor age), graft preparation method (surgeon-prepared versus eye bank-prepared), or tamponade agent used (air versus SF6). Previous studies have suggested that these factors may influence endothelial cell survival and corneal hydration control, which could in turn affect epithelial thickness patterns [44,45]. In our cohort, all grafts were surgeon-prepared on the day of surgery from donors with endothelial cell density exceeding 2200 cells/mm, and 50% SF6 was used as tamponade in all cases, but we did not analyse variations in these parameters. Ninth, we did not evaluate corneal biomechanical properties or correlate them with epithelial thickness patterns. Recent studies have demonstrated relationships between corneal biomechanics and epithelial thickness distribution, particularly in conditions like keratoconus [46]. Understanding whether post-DMEK biomechanical changes contribute to the altered I–S gradient would require dedicated biomechanical assessments using technologies such as the Corvis ST or Brillouin microscopy. Finally, we did not assess corneal innervation status in our patients. Given the important role of corneal nerves in regulating epithelial proliferation, migration, and metabolism, differences in nerve regeneration patterns between superior and inferior cornea could contribute to the observed epithelial thickness gradient. In vivo confocal microscopy studies of corneal nerve density after DMEK would provide valuable insights into the potential contribution of neural factors to epithelial remodelling [33,34].

Future research should address these limitations through several approaches. First, prospective longitudinal studies with serial epithelial thickness measurements beginning preoperatively and continuing through extended postoperative follow-up would elucidate the time course and mechanisms of epithelial remodelling after DMEK. Second, correlation of epithelial thickness patterns with visual outcomes, aberrometry data, contrast sensitivity, and patient-reported visual quality measures would clarify the functional significance of epithelial changes. Third, investigation of factors influencing epithelial remodelling‚ including donor characteristics, surgical technique variations, and individual patient factors such as diabetes or dry eye disease‚ would enable identification of modifiable factors to optimise outcomes. Fourth, studies including patients across all FED severity stages, including those with preoperative epithelial oedema, would provide a comprehensive understanding of epithelial recovery patterns across different clinical scenarios. Fifth, biomechanical assessment using multiple complementary technologies (Corvis ST, Brillouin microscopy, elastography) combined with epithelial thickness mapping would elucidate mechanistic relationships between corneal biomechanics and epithelial architecture after DMEK. Sixth, comparative studies of epithelial changes after DMEK versus DSAEK and penetrating keratoplasty would determine whether the augmented I–S gradient is specific to DMEK or represents a common feature of endothelial keratoplasty. Given that DMEK involves replacing a thinner tissue layer than DSAEK, and that penetrating keratoplasty replaces all corneal layers, comparing epithelial patterns across these techniques could provide insights into the relative contributions of stromal versus endothelial factors to epithelial remodelling. Seventh, the investigation of epithelial thickness patterns in eyes with DMEK graft failure or chronic endothelial dysfunction would determine whether epithelial mapping can serve as a biomarker for graft health. Prospective monitoring of epithelial thickness changes preceding clinical decompensation could establish thresholds for the early detection of failing grafts. Finally, molecular and histopathologic studies examining growth factor expression, basement membrane structure, and keratocyte–epithelial interactions in post-DMEK corneas would provide mechanistic insights into the cellular processes governing epithelial remodelling.

This study demonstrates that DMEK effectively restores CET to values comparable to normal eyes in patients with Stage 2 and 3 FED, while significantly accentuating the physiologic inferior–superior epithelial gradient. Post-DMEK corneas exhibit slightly lower CCTs than controls, likely reflecting chronic stromal remodelling, although the age difference between groups warrants consideration. The pattern of thicker inferior corneal epithelium compared to the superior is preserved and enhanced in post-DMEK patients. Importantly, while a statistically significant but clinically negligible correlation between age and CET was observed in the post-DMEK group, age does not meaningfully influence epithelial thickness distribution in either group. These findings contribute to our understanding of corneal wound healing and remodelling after endothelial keratoplasty and establish normative epithelial thickness patterns for post-DMEK eyes.

## Figures and Tables

**Table 1:** Summary of the patients recruited in this study.

Variable	Post-DMEK Eyes	Control Eyes
Number of eyes	36	36
Sex (female, %)	61	58
Age (years), mean ± SD	71.4 ± 12.1	45.5 ± 14.6
Central epithelial thickness (µm)	53.7 ± 5.5	52.7 ± 3.3
Superior epithelial thickness 2–7 mm (µm)	50 ± 6	52 ± 4
Inferior epithelial thickness 2–7 mm (µm)	56 ± 6	54 ± 4
I–S epithelial thickness difference (µm)	5.9 ± 4.3	3.0 ± 2.2
Central corneal thickness (µm)	525.7 ± 98.4	544.7 ± 27.8
Follow-up after DMEK (months), median (IQR)	24 months (IQR: 12–54 months	NA

SD = standard deviation; I–S = inferior–superior; DMEK = Descemets membrane endothelial keratoplasty; IQR = interquartile range; NA = not applicable.

## Data Availability

Dataset available on request from the authors.

## Supplementary Materials

The following supporting information can be downloaded at: https://www.mdpi.com/article/10.3390/jcm15051984/s1, Supplementary Figure S1A: Inferior–Superior difference (I–S difference) in epithelial thickness versus age; Supplementary Figure S1B: Inferior–Superior difference (I–S difference) in epithelial thickness versus central corneal thickness (CCT); Supplementary Table S1: Demographics and epithelial thickness of post-DMEK (P) and control (C) patients. Supplementary Table S2: Epithelial parameters stratified by follow-up duration in post-DMEK eyes.

## References

[1] Vanathi M. (2024). Corneal epithelial thickness mapping. Indian J. Ophthalmol..

[2] Torricelli A.A., Singh V., Santhiago M.R., Wilson S.E. (2013). The corneal epithelial basement membrane: Structure, function, and disease. Investig. Ophthalmol. Vis. Sci..

[3] Reinstein D.Z., Archer T.J., Vida R.S. (2022). Applications of epithelial thickness mapping in corneal refractive surgery. Saudi J. Ophthalmol..

[4] Verma N., Khare D., Poe A.J., Amador C., Ghiam S., Fealy A., Ebrahimi S., Shadrokh O., Song X.Y., Santiskulvong C. (2023). MicroRNA and Protein Cargos of Human Limbal Epithelial Cell-Derived Exosomes and Their Regulatory Roles in Limbal Stromal Cells of Diabetic and Non-Diabetic Corneas. Cells.

[5] Reinstein D.Z., Archer T.J., Gobbe M., Silverman R.H., Coleman D.J. (2008). Epithelial thickness in the normal cornea: Three-dimensional display with Artemis very high-frequency digital ultrasound. J. Refract. Surg..

[6] Reinstein D.Z., Yap T.E., Archer T.J., Gobbe M., Silverman R.H. (2015). Comparison of Corneal Epithelial Thickness Measurement Between Fourier-Domain OCT and Very High-Frequency Digital Ultrasound. J. Refract. Surg..

[7] Morgado C.R., Santhiago M.R. (2025). Normative distribution of corneal epithelial thickness on 9-mm OCT maps. Front. Med..

[8] Thaung C., Davidson A.E. (2022). Fuchs endothelial corneal dystrophy: Current perspectives on diagnostic pathology and genetics-Bowman Club Lecture. BMJ Open Ophthalmol..

[9] Ong Tone S., Kocaba V., Bohm M., Wylegala A., White T.L., Jurkunas U.V. (2021). Fuchs endothelial corneal dystrophy: The vicious cycle of Fuchs pathogenesis. Prog. Retin. Eye Res..

[10] Bonanno J.A. (2012). Molecular mechanisms underlying the corneal endothelial pump. Exp. Eye Res..

[11] Adamis A.P., Filatov V., Tripathi B.J., Tripathi R.C. (1993). Fuchs’ endothelial dystrophy of the cornea. Surv. Ophthalmol..

[12] Waring G.O., Bourne W.M., Edelhauser H.F., Kenyon K.R. (1982). The corneal endothelium. Normal and pathologic structure and function. Ophthalmology.

[13] Hernandez-Quintela E., Mayer F., Dighiero P., Briat B., Savoldelli M., Legeais J.M., Renard G. (1998). Confocal microscopy of cystic disorders of the corneal epithelium. Ophthalmology.

[14] Wilhelm T.I., Gauche L., Bohringer D., Maier P., Heinzelmann S., Glegola M., Kammrath Betancor P., Reinhard T. (2025). Ten-year outcomes after DMEK, DSAEK, and PK: Insights on graft survival, endothelial cell density loss, rejection and visual acuity. Sci. Rep..

[15] Patel S.V. (2012). Graft survival and endothelial outcomes in the new era of endothelial keratoplasty. Exp. Eye Res..

[16] Arnalich-Montiel F., Mingo-Botin D., Diaz-Montealegre A. (2019). Keratometric, Pachymetric, and Surface Elevation Characterization of Corneas With Fuchs Endothelial Corneal Dystrophy Treated With DMEK. Cornea.

[17] Machalinska A., Kuligowska A., Kaleta K., Kusmierz-Wojtasik M., Safranow K. (2021). Changes in Corneal Parameters after DMEK Surgery: A Swept-Source Imaging Analysis at 12-Month Follow-Up Time. J. Ophthalmol..

[18] Storp J.J., Lahme L., Al-Nawaiseh S., Eter N., Alnawaiseh M. (2023). Descemet Membrane Endothelial Keratoplasty (DMEK) Reduces the Corneal Epithelial Thickness in Fuchs’ Patients. J. Clin. Med..

[19] Alghamdi A., Khan M.S., Dakhil T.A. (2022). Understanding Corneal Epithelial Thickness Mapping. Middle East Afr. J. Ophthalmol..

[20] Satue M., Idoipe M., Gavin A., Romero-Sanz M., Liarakos V.S., Mateo A., Garcia-Martin E., Blasco-Martinez A., Sanchez-Perez A. (2018). Early Changes in Visual Quality and Corneal Structure after DMEK: Does DMEK Approach Optical Quality of a Healthy Cornea?. J. Ophthalmol..

[21] Rocha K.M., Perez-Straziota C.E., Stulting R.D., Randleman J.B. (2013). SD-OCT analysis of regional epithelial thickness profiles in keratoconus, postoperative corneal ectasia, and normal eyes. J. Refract. Surg..

[22] Elhalis H., Azizi B., Jurkunas U.V. (2010). Fuchs endothelial corneal dystrophy. Ocul. Surf..

[23] Mukhija R., Quiney G., Nanavaty M.A. (2023). Clinical Outcomes of Descemet’s Membrane Endothelial Keratoplasty without Routine Prophylactic Peripheral Iridotomy. Vision.

[24] Chew F.M., Teeluck K., Nanavaty M.A. (2018). A paracentesis to save time and money with re-bubbling after descemets membrane endothelial keratoplasty. Eye.

[25] Zhang J., Patel D.V. (2015). The pathophysiology of Fuchs’ endothelial dystrophy--a review of molecular and cellular insights. Exp. Eye Res..

[26] Verma N., Arora S., Singh A.K., Kumar A. (2025). Extracellular Vesicle-Associated miRNAs in Cornea Health and Disease: Diagnostic Potential and Therapeutic Implications. Targets.

[27] Hayashi T., Kobayashi A., Takahashi H., Oyakawa I., Kato N., Yamaguchi T. (2020). Optical characteristics after Descemet membrane endothelial keratoplasty: 1-year results. PLoS ONE.

[28] Abtahi M.A., Beheshtnejad A.H., Latifi G., Akbari-Kamrani M., Ghafarian S., Masoomi A., Sonbolastan S.A., Jahanbani-Ardakani H., Atighechian M., Banan L. (2024). Corneal Epithelial Thickness Mapping: A Major Review. J. Ophthalmol..

[29] Tana-Rivero P., Orts-Vila P., Tana-Sanz P., Ramos-Alzamora M., Montes-Mico R. (2024). Assessment of corneal epithelial thickness mapping by spectral-domain optical coherence tomography. Front. Med..

[30] Kanellopoulos A.J., Asimellis G. (2014). Corneal epithelial remodeling following cataract surgery: Three-dimensional investigation with anterior-segment optical coherence tomography. J. Refract. Surg..

[31] Ghafar N.A., Jalil N.A.A., Kamarudin T.A. (2021). Wound healing of the corneal epithelium: A review. Asian Biomed. (Res. Rev. News).

[32] Gong J., Ding G., Hao Z., Li Y., Deng A., Zhang C. (2024). Elucidating the mechanism of corneal epithelial cell repair: Unraveling the impact of growth factors. Front. Med..

[33] Bandeira F., Yusoff N.Z., Yam G.H., Mehta J.S. (2019). Corneal re-innervation following refractive surgery treatments. Neural Regen. Res..

[34] Pan Q., Angelina A., Marrone M., Stark W.J., Akpek E.K. (2017). Autologous serum eye drops for dry eye. Cochrane Database Syst. Rev..

[35] Pilger D., von Sonnleithner C., Bertelmann E., Maier A.B., Joussen A.M., Torun N. (2018). Exploring the precision of femtosecond laser-assisted descemetorhexis in Descemet membrane endothelial keratoplasty. BMJ Open Ophthalmol..

[36] Colakoglu A., Cosar C.B. (2022). Age-Related Changes in Corneal Epithelial Thickness Measured with an Ultrasound Pachymeter. Clin. Interv. Aging.

[37] Wilson S.E., Marino G.K., Torricelli A.A.M., Medeiros C.S. (2017). Injury and defective regeneration of the epithelial basement membrane in corneal fibrosis: A paradigm for fibrosis in other organs?. Matrix Biol..

[38] Xu M., Yang F., Pazo E.E., Li Q., Yang Z., Huang Y., Zhao S. (2025). Effects of corneal epithelial remodeling on corneal asphericity after FS-LASIK and Trans-PRK: A prospective study. Indian J. Ophthalmol..

[39] Zhu M., Xin Y., Vinciguerra R., Wang Z., Warsame A.M., Wang C., Zhu D., Qu Z., Wang P., Zheng X. (2023). Corneal Epithelial Remodeling in a 6-Month Follow-up Period in Myopic Corneal Refractive Surgeries. J. Refract. Surg..

[40] Bichet P., Moskwa R., Goetz C., Zevering Y., Vermion J.C., Perone J.M. (2023). Five-year clinical outcomes of 107 consecutive DMEK surgeries. PLoS ONE.

[41] Dryander S.E., Volpe-Waizel M.D., Trouvain A.M., Darwisch W., Englisch C.E., Menkene L.M., Szurman P., Boden K., Seitz B., Fries F. (2025). Survival of Grafts up to 10 years After Descemet Membrane Endothelial Keratoplasty. Cornea.

[42] Rothenbuehler S.P., Malmqvist L., Belmouhand M., Bjerager J., Maloca P.M., Larsen M., Hamann S. (2022). Comparison of Spectral-Domain OCT versus Swept-Source OCT for the Detection of Deep Optic Disc Drusen. Diagnostics.

[43] Tan B., Chua J., Harish T., Lau A., Gan A.T.L., Tan Y.L., Wong D.W.K., Chong R.S., Ang M., Husain R. (2020). Comparison of a commercial spectral-domain OCT and swept-source OCT based on an angiography scan for measuring circumpapillary retinal nerve fibre layer thickness. Br. J. Ophthalmol..

[44] Inoda S., Hayashi T., Takahashi H., Oyakawa I., Yokogawa H., Kobayashi A., Kato N., Kawashima H. (2020). Factors associated with endothelial cell density loss post Descemet membrane endothelial keratoplasty for bullous keratopathy in Asia. PLoS ONE.

[45] Romano V., Kazaili A., Pagano L., Gadhvi K.A., Titley M., Steger B., Fernandez-Vega-Cueto L., Meana A., Merayo-Lloves J., Diego P. (2022). Eye bank versus surgeon prepared DMEK tissues: Influence on adhesion and re-bubbling rate. Br. J. Ophthalmol..

[46] Li Y., Xu Z., Liu Q., Wang Y., Lin K., Xia J., Chen S., Hu L. (2021). Relationship between corneal biomechanical parameters and corneal sublayer thickness measured by Corvis ST and UHR-OCT in keratoconus and normal eyes. Eye Vis..

