# Supplementary material for: Epithelial Thickness Changes After Descemet Membrane Endothelial Keratoplasty (DMEK): An Observational Study
Source: J Clin Med. 2026 Mar 5;15(5):1984. doi: 10.3390/jcm15051984 (PMC12985463; doi:10.3390/jcm15051984)
Supplement: Supplementary file 1 [file jcm-15-01984-s001.zip › Supplementary Tabe S1.pdf]

Table S1 - demographics and epithelial thickness of post-DMEK (P) and control (C) patients

	Sex	Age	Eye	Epithelial statistics Superior (S) (2-7mm)	Inferior (I) (2-7 mm)	I-S	Central Epithelial Thickness	CCT	FU
**Cases**									
P1	F	72	OD	50	55	5	52	510	26
P2	M	76	OD	58	62	4	60	522	29
P3	F	79	OS	46	55	9	53	531	29
P4	M	73	OD	49	57	8	53	544	20
P5	M	87	OS	51	54	3	55	487	22
P6	F	72	OS	53	59	6	56	528	18
P7	F	78	OS	45	49	4	51	478	12
P8	M	56	OD	53	56	3	63	454	8
P9	F	60	OD	53	60	7	54	522	16
P10	M	74	OS	42	57	15	59	556	19
P11	M	64	OS	50	58	8	52	521	6
P12	M	97	OS	40	59	19	45	546	13
P13	M	78	OS	47	53	6	56	508	12
P14	M	44	OS	45	54	9	53	606	11
P15	F	78	OS	46	53	7	51	543	14
P16	F	70	OS	49	54	5	49	589	10
P17	F	86	OS	42	46	4	55	519	15
P18	M	78	OS	47	53	6	40	509	8
P19	M	87	OD	68	70	2	55	487	8
P20	M	68	OS	34	49	15	48	712	6
P21	F	49	OS	47	48	1	53	507	6
P22	F	80	OD	51	58	7	58	476	6
P23	F	69	OS	52	50	-2	48	523	6
P24	F	85	OD	50	53	3	65	506	36
P25	F	85	OS	43	53	10	63	513	22
P26	M	67	OS	47	50	3	51	580	96
P27	F	72	OD	55	55	0	54	509	48
P28	M	64	OD	47	47	0	48	538	21
P29	M	80	OD	44	52	8	55	500	114
P30	F	52	OS	45	49	4	48	520	96
P31	F	63	OD	45	56	11	53	472	96
P32	F	57	OD	53	56	3	65	526	96
P33	F	57	OS	51	57	6	55	544	96
P34	F	63	OD	52	58	6	56	531	106
P35	F	63	OS	53	57	4	57	512	106
P36	F	87	OS	41	45	4	47	502	24
**Controls**									
C1	F	OD	22	49	53	4	49	479	N/A
C2	M	OD	54	52	56	4	55	551	N/A

C3	F	OD	42	51	54	3	51	518	N/A
C4	M	OD	51	48	52	4	52	596	N/A
C5	F	OD	28	50	53	3	53	563	N/A
C6	F	OD	54	54	58	4	56	566	N/A
C7	F	OD	35	57	55	-2	55	524	N/A
C8	F	OD	47	54	57	3	55	534	N/A
C9	F	OD	39	48	54	6	51	540	N/A
C10	M	OD	61	50	50	0	47	546	N/A
C11	M	OD	42	52	56	4	55	518	N/A
C12	M	OD	25	54	58	4	56	548	N/A
C13	M	OD	42	51	53	2	53	N/A	N/A
C14	F	OD	86	52	60	8	58	553	N/A
C15	M	OD	55	50	54	4	54	567	N/A
C16	M	OD	33	50	55	5	51	538	N/A
C17	F	OD	42	55	56	1	54	565	N/A
C18	M	OS	62	48	51	3	50	493	N/A
C19	F	OS	22	47	51	4	47	481	N/A
C20	M	OS	54	50	52	2	46	554	N/A
C21	F	OS	42	49	51	2	52	521	N/A
C22	M	OS	51	52	55	3	54	594	N/A
C23	F	OS	28	50	49	-1	49	556	N/A
C24	F	OS	54	54	57	3	54	583	N/A
C25	F	OS	47	51	56	5	53	533	N/A
C26	F	OS	39	48	50	2	48	541	N/A
C27	F	OS	45	53	55	2	56	574	N/A
C28	M	OS	61	50	51	1	47	544	N/A
C29	M	OS	42	52	56	4	56	526	N/A
C30	M	OS	25	54	58	4	58	554	N/A
C31	M	OS	42	53	55	2	55	518	N/A
C32	F	OS	86	49	58	9	58	550	N/A
C33	M	OS	55	54	54	0	53	574	N/A
C34	M	OS	33	52	56	4	52	534	N/A
C35	F	OS	42	54	55	1	55	560	N/A
C36	M	OS	62	48	51	3	52	N/A	N/A

FED - Fuch's endothelial dystrophy, PK – Penetrating keratoplasty, PBK – Pseudophakic bollus keratopathy, CCT – Central corneal thickness, FU -Follow up, N/A – Non Available
