# Supplementary material for: Epithelial Thickness Changes After Descemet Membrane Endothelial Keratoplasty (DMEK): An Observational Study
Source: J Clin Med. 2026 Mar 5;15(5):1984. doi: 10.3390/jcm15051984 (PMC12985463; doi:10.3390/jcm15051984)
Supplement: Supplementary file 1 [file jcm-15-01984-s001.zip › Supplementary Table S2.pdf]

Supplementary Table S2. Epithelial parameters stratified by follow-up duration in post-DMEK eyes

**Follow-up group**	**CET, mean ± SD (µm)**	**I–S difference, mean ± SD (µm)**	**CCT, mean ± SD (µm)**
6–12 months	52.2 ± 5.8	5.3 ± 4.4	530.8 ± 72.1
12–36 months	54.4 ± 5.7	6.9 ± 4.9	524.5 ± 19.0
>36 months	54.9 ± 4.7	5.0 ± 3.2	521.6 ± 30.0

CET= central epithelial thickness; I-S difference= inferior-superior difference; CCT = central corneal thickness; SD = standard deviation; DMEK = Descemet's membrane endothelial keratoplasty.
