# Supplementary figures and images for: Epithelial Thickness Changes After Descemet Membrane Endothelial Keratoplasty (DMEK): An Observational Study
Source: J Clin Med. 2026 Mar 5;15(5):1984. doi: 10.3390/jcm15051984 (PMC12985463; doi:10.3390/jcm15051984)

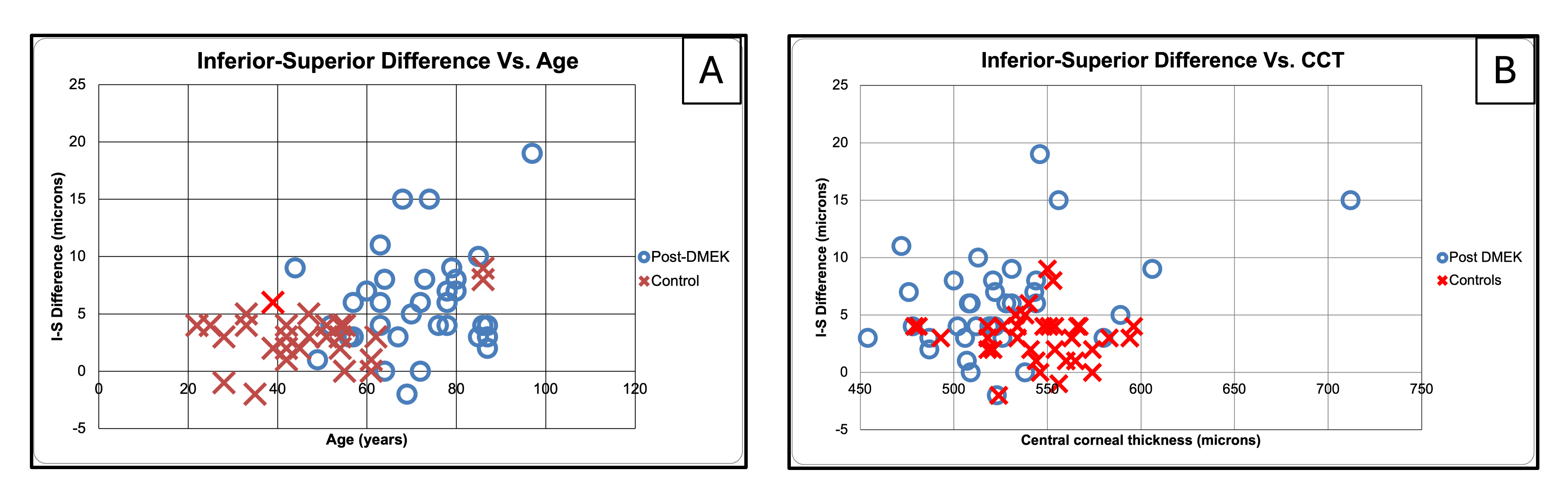

Supplement: Supplementary file 1 [file jcm-15-01984-s001.zip › Supplementary Figure S1A,B.tiff]
